# A drastic superoxide-dependent oxidative stress is prerequisite for the down-regulation of IRP1: Insights from studies on SOD1-deficient mice and macrophages treated with paraquat

**DOI:** 10.1371/journal.pone.0176800

**Published:** 2017-05-19

**Authors:** Anna Milczarek, Rafał R. Starzyński, Agnieszka Styś, Aneta Jończy, Robert Staroń, Agnieszka Grzelak, Paweł Lipiński

**Affiliations:** 1 Department of Molecular Biology, Institute of Genetics and Animal Breeding, Polish Academy of Sciences, Jastrzębiec, Poland; 2 Department of Molecular Biophysics, University of Łódź, Łódź, Poland; CINVESTAV-IPN, MEXICO

## Abstract

Iron regulatory protein 1 (IRP1) is a cytosolic bifunctional [4Fe-4S] protein which exhibits aconitase activity or binds iron responsive elements (IREs) in untranslated regions of specific mRNA encoding proteins involved in cellular iron metabolism. Superoxide radical (O_2_^.-^) converts IRP1 from a [4Fe-4S] aconitase to a [3Fe-4S] „null” form possessing neither aconitase nor *trans*-regulatory activity. Genetic ablation of superoxide dismutase 1 (SOD1), an antioxidant enzyme that acts to reduce O_2_^**.**-^ concentration, revealed a new O_2_^**.**-^-dependent regulation of IRP1 leading to the reduction of IRP1 protein level and in consequence to the diminution of IRP1 enzymatic and IRE-binding activities. Here, we attempted to establish whether developmental changes in SOD1 activity occurring in the mouse liver, impact IRP1 expression. We show no correlation between hepatic SOD1 activity and IRP1 protein level neither in pre- nor postnatal period probably because the magnitude of developmental fluctuations in SOD1 activity is relatively small. The comparison of SOD1 activity in regards to IRP1 protein level in the liver of threeSOD1 genotypes (*Sod1*^*+/+*^, *Sod1*^*+/-*^ and *Sod1*^*-/-*^) demonstrates that only drastic SOD1 deficiency leads to the reduction of IRP1 protein level. Importantly, we found that in the liver of fetuses lacking SOD1, IRP1 is not down-regulated. To investigate O_2_^**.**-^-dependent regulation of IRP1 in a cellular model, we exposed murine RAW 264.7 and bone marrow-derived macrophages to paraquat, widely used as a redox cycler to stimulate O_2_^**.**-^production in cells. We showed that IRP1 protein level as well as aconitase and IRE-binding activities are strongly reduced in macrophages treated with paraquat. The analysis of the expression of IRP1-target genes revealed the increase in L-ferritin protein level resulting from the enhanced transcriptional regulation of the *LFt* gene and diminished translational repression of L-ferritin mRNA by IRP1. We propose that O_2_^**.**-^-dependent up-regulation of this cellular protectant in paraquat-treated macrophages may counterbalance iron-related toxic effects of O_2_^**.**-^.

## Introduction

Cellular iron homeostasis has to complete two major biological tasks: (i) ensure the availability of iron for fundamental metabolic processes; (ii) minimize the ability of the metal to catalyze the formation of highly toxic hydroxyl radical through the Fenton reaction. These processes are largely controlled by the post-transcriptional IRP/IRE regulatory system. Iron regulatory proteins (IRP1 and IRP2) are cytoplasmic proteins that play a critical role in this regulation by interacting with mRNA hairpin structures called iron responsive elements (IREs). These elements are present in the untranslated regions (UTR) of mRNAs encoding subunits of iron storage protein, ferritin (L- and H-Ft) and in both iron transporters: transferrin receptor 1(TfR1) and ferroportin (Fpn) involved in iron import and export, respectively [1]. The binding of IRPs to the unique IRE in the 5’-UTR of L- and H-Ft mRNAs blocks the translation initiation by preventing the association of 43S translation pre-initiation complex. In contrast, the binding of IRPs to IREs in the 3’-UTR of TfR1 mRNA is thought to protect this mRNA against degradation by preventing access of a nuclease, whose cleavage site is close to the IREs [2]. IRP1 is a bifunctional protein showing either aconitase or *trans*-regulatory activity. Both IRP1 activities are mutually exclusive depending on the presence or absence of the [4Fe-4S] cluster [3]. In iron-replete cells, IRP1 assembles an iron-sulfur [4Fe-4S] cluster and functions as a cytosolic aconitase able to convert citrate to *iso*-citrate. Under iron-deficient conditions, IRP1 accumulates as an apo-form, lacking the [4Fe-4S] cluster, and gains the ability to recognize IREs with high affinity. The coordinated bi-directional regulation of Ft, TfR1 and Fpn mRNAs by IRPs allows rapid changes in gene expression in response to iron fluctuations, and ensures that the cells acquire sufficient iron for their requirement while preventing iron toxicity. Studies on mice with targeted deletion of IRP1 and IRP2 revealed that the later regulator is critical for maintaining the iron balance *in vivo* [4–6]. On the other hand, there is growing evidence that IRP1 is a molecular target responding preferentially to reactive nitrogen and oxygen species. Indeed, IRP1 is a redox-sensitive gene *trans*-regulator and its [4Fe-4S] cluster located at the critical allosteric site of the enzyme is a crucial component of the cellular response to nitric oxide (NO) [7], peroxynitrite (ONOO^-^) [8], superoxide radical (O_2_^**.**-^) [9], and hydrogen peroxide (H_2_O_2_) [10,11]. Apart from the regulation of IRP1 activities by the post-translational mechanism(s) underlying interactions with its [4Fe-4S] cluster, it is known that NO [12,13] and O_2_^**.**-^ -dependent oxidative stress [14] down-regulate expression of the *Irp1* gene. Resulting decrease in intracellular IRP1 protein level leads to the reduction of its enzymatic and *trans*-regulatory activity [12–14].

We have previously reported that targeted deletion of superoxide dismutase 1 (SOD1; Cu,Zn-SOD), an enzyme that, leads to a drastic down-regulation of IRP1 protein level (close to the total deficiency) in the liver of adult mice [14]. Here, we ask the question whether changes in SOD1 activity naturally occurring during mouse prenatal and postnatal development impact IRP1 level in the liver. We also, investigated the effect of O_2_^**.**-^ on the expression of the *Irp1* gene in a cellular model, i.e. RAW 264.7 macrophages exposed to paraquat (PQ), a redox cycler stimulating O_2_^**.**-^ production [15]. Finally, we use primary cultures of mouse bone marrow-derived macrophages (BMDM), both wild-type (w-t, *Irp1*^*+/+*^) and lacking IRP1 (*Irp1*^*-/-*^), to examine the role of^.^O_2_^**.**-^-dependent decline in IRP1 protein level on the regulation of IRP1-target genes. The results show that IRP1 is down-regulated only under conditions of either profound SOD1 deficiency *in vivo*, in postnatal life or in the presence of high PQ concentration in cultured macrophages, which leads to the generation of an intensive O_2_^**.**-^-dependent oxidative stress. Importantly, treatment of BMDM with PQ results in the enhancement of cellular antioxidant response manifesting by increased L-Ft protein level, which is the effect of combined regulations, i.e. transcriptional induction of the *LFt* gene and reduced translational repression of L-Ft transcript under IRP1 scarcity.

## Materials and methods

### Ethical statement

Second (2^nd^) Local Ethical Committee on Animal Testing at the Warsaw University of Life Sciences (SGGW) in Warsaw granted a formal waiver of the ethical approval because the only procedure involved in the study was euthanasia. Animals were euthanized by peritoneal injection of Vetbutal (Biovet, Puławy, Poland) preceeded by sedation with ketamine and xylazine administered intraperitoneally.

### Mice

SOD1 knock-out (*Sod1*^-/-^) mice and the corresponding *Sod1*^*+/-*^ and *Sod1*^*+/+*^ controls were provided by The Jackson Laboratory (Bar Harbor, ME) and were described in details previously [14,16]. For timed matings of *Sod1*^*+/-*^ animals, the morning plug was identified and was considered E0.5. Plugged females were then euthanized at E14.5 and E18.5. Embryos/fetuses were dissected from the uterus, their livers were collected and genotyped. Mice with truncated *Aco1* (herein designated *Irp1*^*-/-*^) allele, have been kindly provided by Drs B. Galy and M.W. Hentze (EMBL, Heidelberg, Germany). IRP-null animals, and their corresponding wild-type littermates (*Irp1*^*+/+*^) were obtained from heterozygous intercrosses. Genotyping of the progeny was performed as previously described [4]. Mice were kept under a constant light/dark cycle on a standard mouse diet.

### Macrophage culture and treatment

RAW 264.7 murine macrophages, a cell line established from a tumour induced by Abelson murine leukaemia virus, were obtained from the American Type Culture Collection (Rockville, MD, U.S.A.). Cells were cultured in DMEM (Biowest) containing 5% (v/v) FCS and gentamicin (50 *μ*g/ml) in 100 cm^2^ plastic culture flasks (Nunc) in a humidified atmosphere of 95% air and 5% CO_2_ at 37°C.

Bone marrow-derived macrophages (BMDM) were isolated from tibia, femur and humerus of 2-month-old *Irp1*^*+/+*^ and *Irp1*^*-/-*^ mice and seeded in 10 cm diameter Petri dishes for RNA and protein extraction. Cells were cultured in RPMI 1640 medium (HyClone) supplemented with 10% heat inactivated FBS (Eurx), 10% LCCM (L929-cell conditioned medium as a source of macrophages colony-stimulating factor) and 1% penicillin/streptomycin (Sigma) at 37°C, in 5% CO_2_ and 21% O_2_ atmosphere. After four days, cells were rinsed three times with PBS and the medium was subsequently replaced every two days until day seven.

The mouse RAW 264.7 cells and BMDM were incubated in medium supplemented with 500 μM paraquat (PQ,1,1′-Dimethyl-4,4′-bipyridinium dichloride hydrate, Sigma-Aldrich), a redox cycler stimulating superoxide production [15], for 2 hours. After the end of exposure to PQ, macrophages were extensively washed and further cultured in a fresh medium as indicated in the figure legends. Control cells were cultured in parallel in the absence of PQ. At the indicated times, cell were harvested, and both cytosol and mitochondria-enriched fractions were prepared [7]. To determine the expression of IRP1, L-Ft and TfR1 mRNAs, total RNAs were extracted in parallel.

### Measurement of superoxide dismutase activity

SOD activity in hepatic and renal cytosolic extracts was measured by gel electrophoresis using the NitroblueTetrazolium (NBT)/riboflavin method as described previously [17].

### RNA extraction and real-time quantitative RT-PCR

Total RNA was extracted from BMDM or livers by using the High Pure RNA Isolation and High Pure RNA Tissue kits (Roche Diagnostics), respectively. Total RNA (1 *μ*g) was reverse transcribed with random hexamers using Transcriptor First Strand cDNA Synthesis Kit (Roche Diagnostics). IRP1 and TfR1 mRNAs levels were measured by real-time quantitative RT-PCR as described previously [16]. Specific cDNA fragments were amplified using the following pairs of oligonucleotide primers: IRP1, 5’-TCC ACC ACC CTG TTG CTG TAG-3’ (forward) and 5′-GCG TCG AAT ACA TCA AGG GT-3′ (reverse); L-Ft, 5′-CGG AGG GTC AAC ATG CTA TAA-3′ (forward) and 5′-AAG AGA CGG TGC AGA CTG GT-3′ (reverse); TfR1, 5′-TGC AGC AGC TCT TGA GAT TG-3′ (forward) and 5′-GTT GAG GCA GAC CTT GCA CT-3′ (reverse). The reactions were performed in a Light Cycler (Roche Diagnostics) and Light Cycler 3.5 Software was used for data analysis. Expression was quantified relative to that of control transcripts encoding glyceraldehyde 3-phosphate dehydrogenase (GAPDH), 5’-GAC CAC AGT CCA TGC CAT CAC-3’ (forward) 5’-TCC ACC ACC CTG TTG CTG TAG-3’ and18 S ribosomal RNA, 5′-CTG AGA AAC GGC TAC CAC ATC-3′ (forward) and 5′-CGC TCC CAA GAT CCA ACT AC-3′ (reverse).

### Immunoblot analysis

For the detection of liver, kidney and macrophage IRP1 and macrophage L-ferritin subunit 50 *μ*g of respective cytosolic extracts (prepared as described previously [14]) were resolved by electrophoresis on 8%and 15% SDS/PAGE gels, respectively. SOD1 was detected in total protein extracts obtained from tissues. Electroblotting of resolved proteins on to a PVDF membrane (Millipore), blocking and incubation with primary antibodies was performed as described previously [16]. The following primary antibodies were used: a chicken polyclonal antibody raised against purified human recombinant IRP1 (Agro-Bio, La Ferté Saint-Aubin, France), and rabbit antisera raised against L (light chain)-Ft (provided by Dr. P. Santambrogio, San Raffaele Scientific Institute, Milan, Italy) rabbit polyclonal anti-superoxide dismutase 1 antibody (Abcam ab16831). Membranes were then washed and incubated with peroxidase-conjugated anti-chicken or anti-rabbit secondary antibodies (Santa Cruz Biotechnology) for 1 h at room temperature (20°C). Immunoreactive bands were detected using the ECL (enhanced chemiluminescence) Plus Western blotting detection system (Amersham Life Sciences). Quantification was performed relative to β-actin detected using a specific antibody against mouse actin (Santa Cruz Biotechnology) using a Molecular Imager with Quantity One software (Bio-Rad).

### Measurement of IRP1 activities

IRP1 aconitase activity in liver cytosolic extracts was measured spectrophotometrically by following the disappearance of *cis*-aconitate at 240 nm at 37°C, as described previously [18]. IRP1-IRE interactions were examined as described previously [19] by incubating 2 μg of the cytosolic protein extracts with a molar excess of [^32^P]CTP-labeled H-ferritin IRE probe. In parallel experiments, cytosolic extracts were treated with 2-mercaptoethanol at a final concentration of 2% before the addition of the IRE probe, to produce maximal IRE-binding activity [20]. IRE-protein complexes were then separated by electrophoresis on 6% non-denaturing polyacrylamide gels. The signals representing the IRE-IRP1 complexes were quantified with a Molecular Imager using Quantity One software (Bio-Rad).

### Statistical methods

Statistical analysis was performed using Statistica 12 software. We determined significance by unpaired two-tailed Student’s *t* test to asses data, with *p* values of <0.05 and <0.01 being considered statistically significant and highly significant, respectively.

## Results

### Drastic decrease in SOD1 activity is prerequisite for down-regulation of IRP1 protein level in the mouse liver and kidney

**Fig 1:**
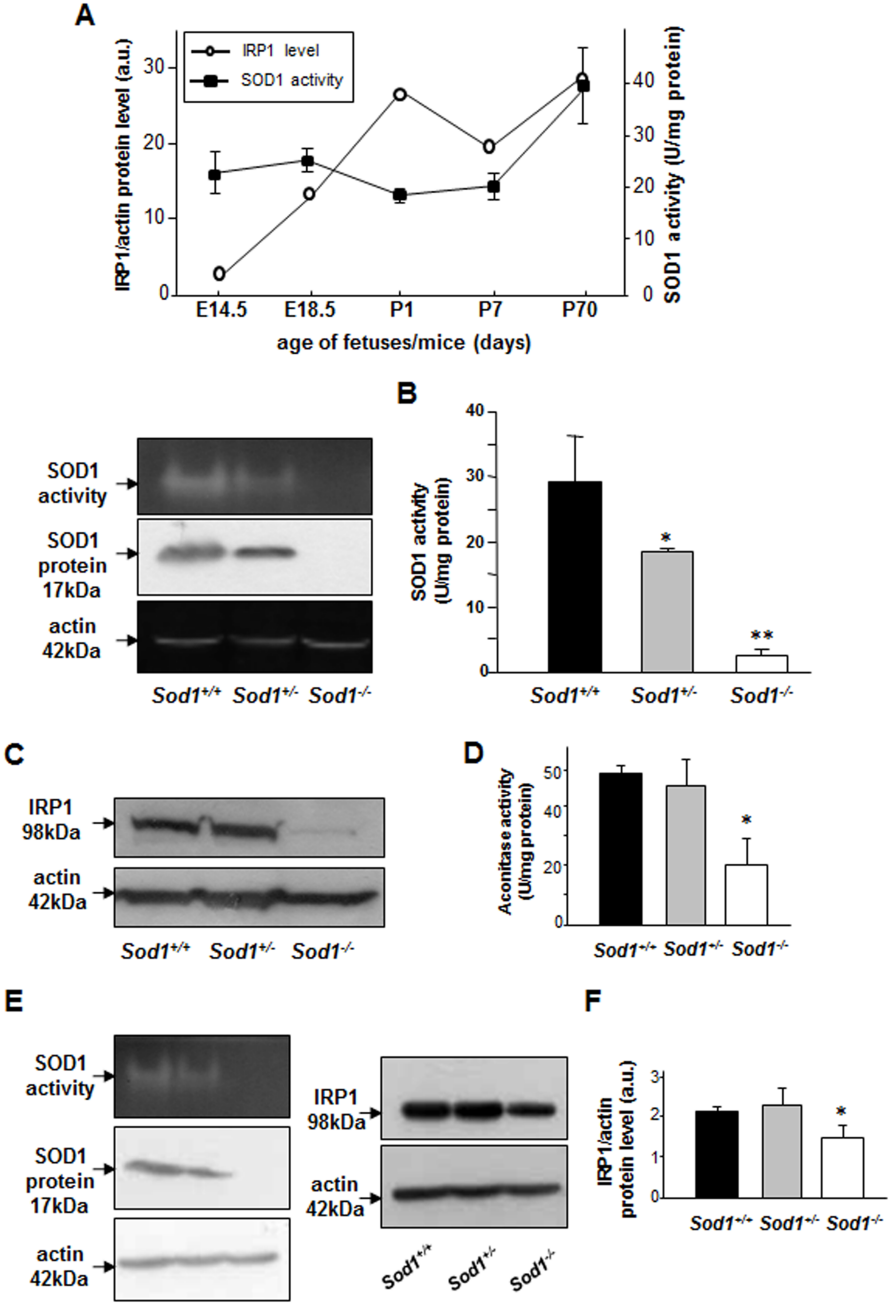
Hepatic SOD1 activity and IRP1 protein level are not correlated during mouse development. Drastic decline in SOD1 activity is mandatory for the down-regulation of IRP1 in the mouse liver and kidney. **(A)** SOD1 activity and IRP1 protein level in prenatal and postnatal periods. For the measurements in prenatal period cytosolic extracts were prepared from pooled fetal livers obtained from 4–5 fetuses at the given age. Data are representative for 2 sets of pooled fetal liver samples obtained from 2 pregnant females. Results of hepatic SOD1 activity and IRP1 protein level in postnatal period were obtained from analyses performed on liver samples collected from 3 separate mice. **(B**) *left-hand panel*, hepatic activity and protein level of SOD1in mice of 3 SOD1 genotypes (aged 2 months). *right-hand panel*, the intensity of the SOD1activity bands was quantified with a molecular Imager using Quantity One software (Bio-Rad) and plotted in arbitrary units to present enzyme activity. Results are expressed as mean ± S.D. for 5 mice of each genotype. **(C)** hepatic IRP1 protein level in mice of 3 SOD1 genotypes. *right-hand panel*, the intensity of the IRP1 bands was quantified with a molecular Imager using Quantity One software (Bio-Rad) and is plotted in arbitrary units to present IRP1 protein level. **(D)** IRP1 aconitase activity determined spectrophotometrically in hepatic cytosolic extracts by measuring the disappearance of *cis*-aconitate at 240 nm as described previously [19]. **(E)** renal activity and protein level of SOD1 in mice of 3 SOD1 genotypes (aged 2 months). **(F)** *left-hand panel*, renal IRP1 protein level in mice of 3 SOD1 genotypes. *right-hand panel*, the intensity of the IRP1 bands was quantified with a molecular Imager using Quantity One software (Bio-Rad) and is plotted in arbitrary units to present IRP1 protein level. Results in **(C), (D), (E)** and **(F)** are expressed as mean ± S.D. for 3 2-month old mice of each genotype. Statistically significant differences are indicated (*P<0.05; **P<0.01).

Using wild-type fetuses and mice we aimed to test the hypothesis that IRP1 protein level in the liver during mouse development is associated with changes in SOD1 activity. We therefore measured both hepatic SOD1 activity and IRP1 level in fetuses on days E14.5 and E18.5 of prenatal period and in mice on days 1, 7 and 70 after birth. Our results clearly show that developmental changes in SOD1 activity do not influence IRP1 level in the liver (Fig 1A). Although during prenatal period changes of hepatic SOD1 activity and IRP1 level are positively correlated, just after the birth (P1) a strong induction of IRP1 is accompanied by the decrease in SOD1 activity. Then, in the postnatal period we reported either negative (P7) or positive correlation between examined parameters. Importantly, during whole examined developmental period SOD1 activities vary in a relatively narrow range of values (~20U/mg protein) whereas IRP1 shows approximately 10-fold increase from E14.5 to P70. The comparison of both parameters in 2-month-old mice of 3 genotypes: wild-type (*Sod1*^*+/+*^), heterozygous (*Sod1*^*+/-*^) and homozygous (*Sod1*^*-/-*^) for the non-functional *SOD1* allele demonstrates that the decrease in hepatic IRP1 protein level was only observed in *Sod1*^*-/-*^ mice showing residual SOD1 activity (Fig 1B and 1C). Decrease in SOD1 activity in *Sod1*^+*/-*^mice (30%) did not result in the reduction of IRP1 level (Fig 1B). Considering the physiological significance of the IRP1-HIF2α axis in regulating erythropoiesis via renal erythropoietin [21–23] we checked whether SOD1 deficiency impacts IRP1 level in the kidney. Similarly to our finding in the liver, we observed that IRP1 protein level is significantly decreased only in the kidney of *Sod1*^*-/-*^ mice although to much lesser extent than in the liver of those animals (Fig 1F). Altogether, our results suggest that only under conditions of drastic decline in SOD1 activity, IRP1 protein level is down-regulated.

### IRP1 level in the liver of *Sod1*^*-/-*^ mice is down-regulated during the postnatal, but not prenatal life

**Fig 2:**
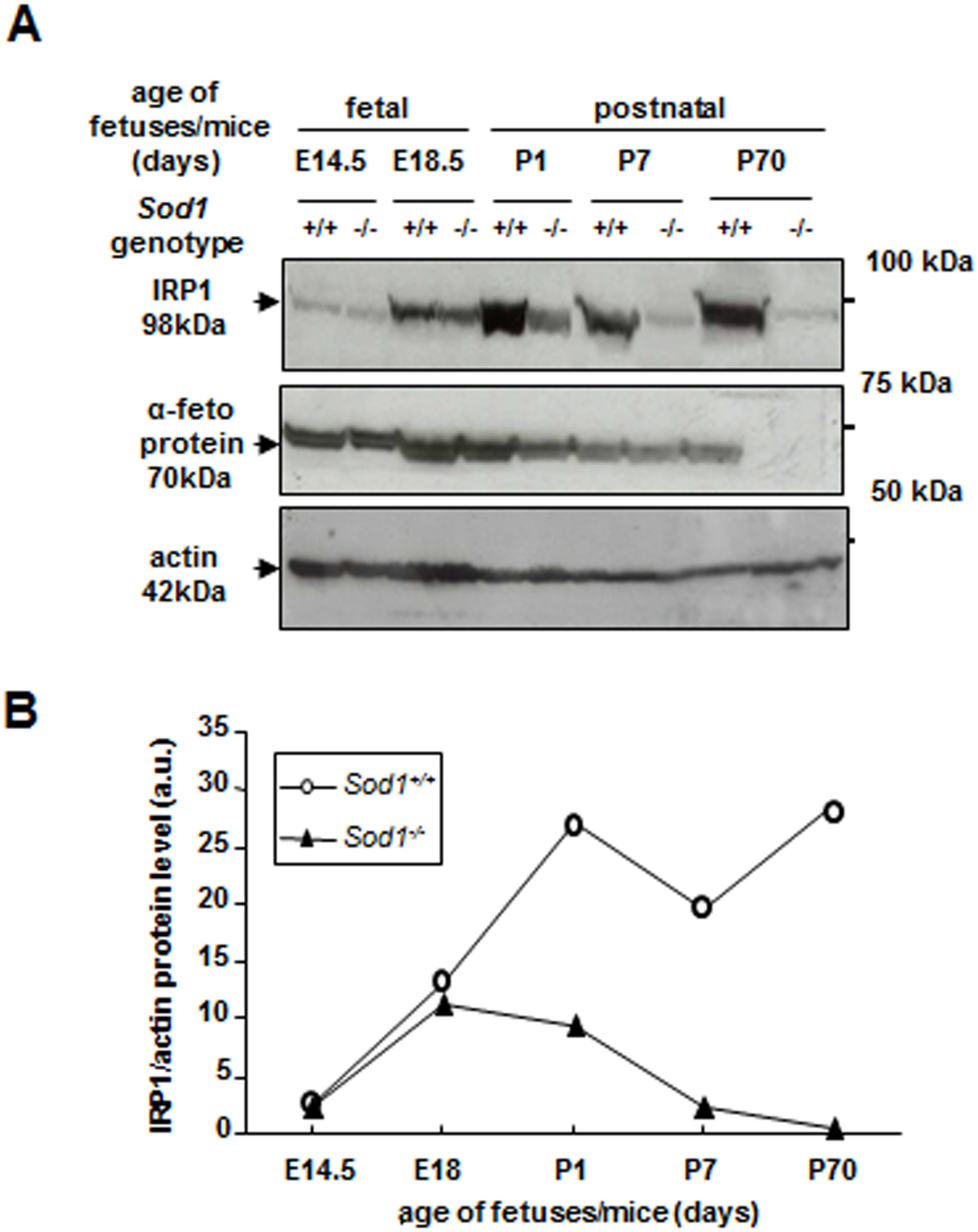
Genetic ablation of SOD1 does not impact hepatic IRP1 protein in fetal livers. For the analysis ofIRP1 protein level in prenatal period cytosolic extracts were prepared from pooled fetal livers obtained from 4–5 fetuses of *Sod1*^*+/+*^ and *Sod1*^*-/-*^ genotypes at the given age. **(A)** Data shown are representative for 2 sets of 4–5 pooled fetal liver samples obtained from 2 pregnant females. Results of hepatic IRP1 protein level in postnatal period are representative of western blot analyses performed on liver samples collected from 3 separate mice of each genotype. Actin was used as a loading control for all samples, but α-fetoprotein was used in addition as a control for fetal livers(antibody raised against recombinant AFP of human origin, which corss-reacts with mouse protein, Santa Cruz Biotechnology) **(B)** The intensity of the IRP1 bands shown in **(A)** was quantified with a molecular Imager using Quantity One software (Bio-Rad) and is plotted in arbitrary units to present IRP1 protein level.

Previously, we showed that the expression of *Irp1* gene was markedly decreased in the liver of adult superoxide dismutase 1 (SOD1) knockout mice [14]. Considering that fetal development proceeds in an environment that is relatively hypoxic, as compared to postnatal oxygen exposure [24,25], we investigated whether IRP1 down-regulation occurs in the *Sod1*^*-/-*^ fetal liver. Protein level of IRP1 was analyzed in livers of E14.5-and E18.5-day old *Sod1*^*+/+*^ and *Sod1*^*-/-*^fetuses and no differences were found (Fig 2). Interestingly, in fetuses of both genotypes hepatic IRP1 showed a marked increase from day E14.5 to day E18.5 of prenatal life. Divergence in hepatic IRP1 level between mice of the two SOD1 genotypes appeared on day 1 of postnatal life, when IRP1 level started to be regulated in an opposite way, i.e. increased in *Sod1*^*+/+*^ and declined in *Sod1*^*-/-*^ mice, respectively. Then, IRP1 level in *Sod1*^*-/-*^mice continued to drop up to day 70 of postnatal period.

### Paraquat (PQ) decreases IRP1 expression and modulates its activities in mouse macrophages

**Fig 3:**
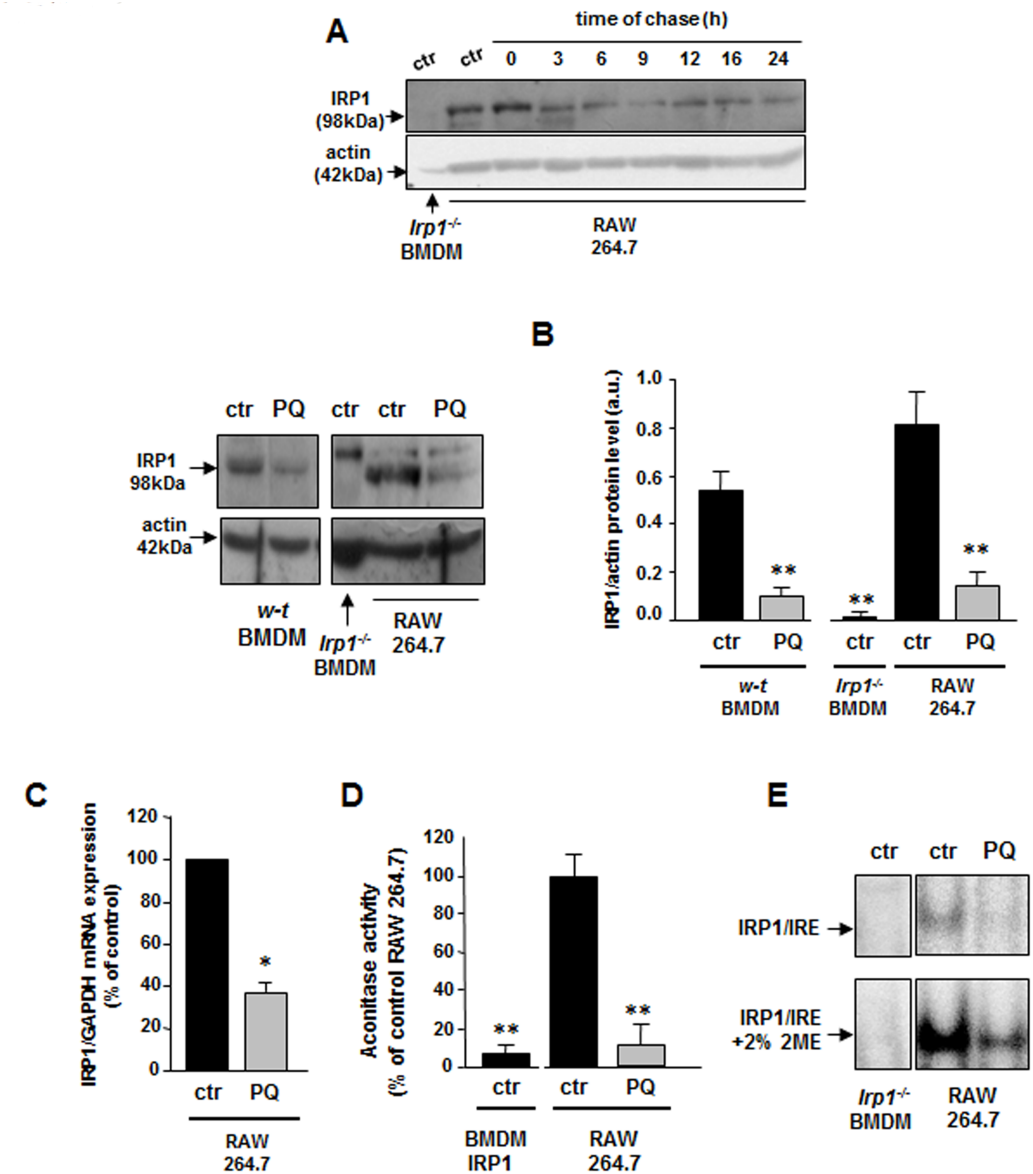
Down-regulation of IRP1 expression and IRP1 activities in mouse macrophages exposed to paraquat (PQ). **(A)** Time course of the modulation of IRP1 protein level in RAW 264.7 macrophages exposed to PQ. RAW 264.7 cells were treated for 2h with 500 μM PQ as described in Materials and methods. At indicated time-points after PQ withdrawal, cells were harvested and cytoslic extracts were prepared as described previously [55]. IRP1 levels were analyzed by Western blotting as described under Materials and methods. The analyses were performed using cell cytosolic extracts obtained from cells from 4 separate experiments, and representative results are shown. **(B)** Down-regulation of IRP1 protein level in mouse bone marrow-derived macrophages and RAW 264.7 cells treated for 2 hours with 500 μM PQ and after its withdrawal cultured for additional 6 h. *left-hand panel*, Representative results of 4 separate biological experiments are shown. *right-hand panel*, the intensity of the IRP1 bands was quantified with a molecular Imager using Quantity One software (Bio-Rad) and is plotted in arbitrary units to present protein level. Results are expressed as mean ± S.D. for 4 separate *in vitro* experiments. **(C)** Decrease in IRP1 mRNA abundance in RAW 264.7 macrophages treated with PQ as described in **(B)**. IRP1 mRNA abundance in cells was measured by real-time RT-PCR as described in Materials and methods. Each column represents the mean (± S.D.) of two amplification reactions, performed on a single cDNA sample reverse-transcribed from RNA prepared from cells from three biological experiments. **(D)** IRP1 aconitase activity (means ± S.D. n = 5 biological experiments) determined spectrophotometrically in hepatic cytosolic extracts by measuring the disappearance of *cis*-aconitate at 240 nm as described previously [12]. **(E)** IRP1 IRE binding activity in response to PQ treatment. Measurements were performedas described under Materials and methods. Data shown are representative of EMSA analyses 4 separate biological experiments. Cytosolic extracts obtained from BMDM lacking IRP1 derived from *Irp1*^*-/-*^ mice were used as negative controls. Statistically significant differences are indicated (*P<0.05; **P<0.01).

Cellular model of genetic SOD1 deficiency, i.e. *Sod1*^*-/-*^ BMDM, are not viable in *in vitro* culture even in the atmosphere of 3% oxygen (O_2_) (our unpublished results). Instead, to increase intracellular steady-state level of this radical [26], in our *in vitro* studies we exposed mouse RAW 264.7 cells and BMDM to PQ, a redox cycling agent widely used to stimulate O_2_^**.**-^ production in cells [15]. Cell viability was affected by prolonged PQ (500 μM) treatment (>2 h), therefore, we incubated cells with PQ for 2 h and after its withdrawal from the culture, cells were chased for the indicated durations in the absence of this redox cycler. We investigated the time course of the effect of PQ on IRP1 expression and reported progressive decrease in IRP1 protein level with the largest decline 6–12 h after removing PQ, which was then followed by slow reconstitution of IRP1 protein although not to the control level (Fig 3A).

On the basis of our kinetic experiment we chose 6h time-point (6 hours after PQ withdrawal) to analyze the influence of PQ on IRP1 level, activities and mRNA. Treatment of RAW 264.7 cells resulted in a concerted down-regulation of IRP1 protein level (20% of control, Fig 3B), mRNA IRP1 expression (40% of control, Fig 3D), aconitase activity (10% of control, Fig 3C), both native and 2%-ME-induced IRP1 IRE-binding activity (Fig 3D). Importantly, the level of aconitase 2, was not down-regulated in RAW 264,7 cells treated with PQ (S1 Fig). We also verified how hydrogen peroxide (H_2_O_2_), an oxidant co-generated with O_2_^**.**-^during cell-mediated redox cycling of PQ [15], regulates IRP1 expression. We found that treatment of cells with exogenous H_2_O_2_ did not affect IRP1 protein level (S2 Fig).

### Paraquat treatment increases L-ferritin mRNA and protein levels in *Irp1*^*+/+*^ and *Irp1*^*-/-*^ BMDM

**Fig 4:**
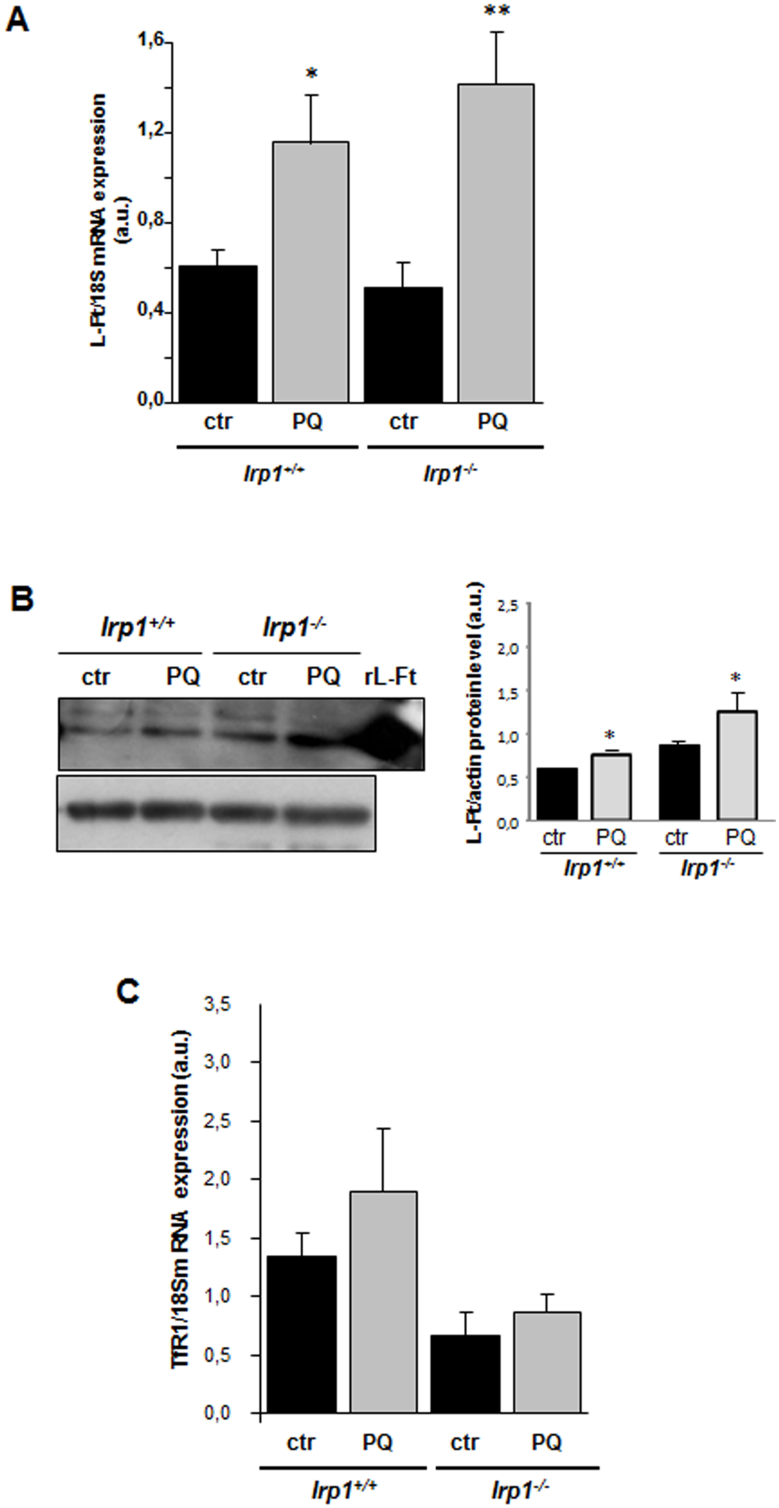
Increase in L-Ft mRNA abundance and L-Ft protein levels in PQ-treated BMDM derived from *Irp1*^*+/+*^ (*wild-type*) and *Irp1*^*-/-*^ mice. BMDM were treated with PQ as described in the legend to Fig 3. **(A)** IRP1 mRNA and **(C)** TfR1 mRNA abundance in BMDM of two genotypes was measured by real-time RT-PCR as described in Materials and methods. Each column represents the mean (± S.D. for PQ-treated RAW 264.7 cells) of two amplification reactions, performed on a single cDNA sample reverse-transcribed from RNA prepared from cells from 5 biological experiments. **(B)** *left-hand panel*, L-Ft levels were analyzed by Western blotting as described in Materials and methods. The analyses were performed using cell cytosolic extracts obtained from cells from 5 separate biological experiments, and representative results are shown. Recombinant mouse L-Ft (rL-Ft), generous gift from Dr. P. Santambrogio, was used as a positive control. *right-hand panel*, the intensity of the L-Ft bands was quantified with a molecular Imager using Quantity One software (Bio-Rad) and is plotted in arbitrary units to present L-Ft protein level. Statistically significant differences are indicated (*P<0.05).

We next investigated whether strong decrease in IRP1 *trans*-regulatory activity in PQ-treated macrophages was followed by a change in the expression of genes whose mRNAs contain an IRE sequence(s) in their 5’- (L-Ft) or 3’-UTR (TfR1). In our experiment we used BMDM derived from *Irp1*^*+/+*^ and *Irp1*^*-/-*^mice. Importantly, IRP1 deficiency itself did not alter the expression of L-Ft at the protein level in intact (non-treated) BMDM (Fig 4B), in accordance with our previous results [27]. However, PQ treatment was found to up-regulate L-Ft mRNA and protein in cells of both IRP1 genotypes to the same extent (Fig 4A). The increase in the L-Ft protein level could be explained by combined effect of transcriptional induction of the *LFt* gene and translational derepression of the L-Ft mRNA resulting from the concomitant PQ-induced down-regulation of IRP1 (*Irp1*^*+/+*^ BMDM) or its constitutive absence (*Irp1*^*-/-*^ BMDM).

In contrast to the *LFt* gene, expression of the *TfR1* seems to depend on IRP1 genotype and is not influenced by PQ treatment. The level of TfR1 mRNA was lower in IRP1-null BMDM compared with wild-type cells either treated or non-treated with PQ (Fig 4C), suggesting that the complete lack of IRP1 partially destabilizes TfR1 transcript. Exposure of BMDM to PQ did not influence TfR1 mRNA expression neither in *Irp1*^*+/+*^ nor *Irp1*^*-/-*^ cells.

## Discussion

Superoxide anion (O_2_^**.**-^) is the product of the one-electron reduction of dioxygen, (O_2_), a biochemical reaction, which occurs widely in nature [26]. This reactive oxygen species (ROS) has been known to be highly toxic to cells for a long time [28], but it has recently become clear that apart from being a harmful product, it also plays a key role in the physiological control of cell function [29]. Due to this biological dichotomy of O_2_^**.**-^, its level in cells needs to be tightly regulated. Superoxide dismutase 1 (SOD1, Cu,Zn-SOD), is a cytosolic member of a superoxide dismutases family of metalloenzymes, that participate in maintaining steady-state O_2_^**.**-^ levels in living cells by catalyzing O_2_^**.**-^ dismutation to hydrogen peroxide (H_2_O_2_) and O_2_ [26]. Permanent oxidative stress associated with genetic *Sod1* deficiency results in an increased incidence of pathological changes, such as hepatocarcinogenesis, hearing loss and muscle atrophy [30–32]. As a consequence, the lifespan of *Sod1* knockout mice is significantly shortened [33]. On the other hand, overexpression of SOD1 disrupts the balance between various ROS, alters redox-sensitive intra- and intercelullar signaling [34] and predisposes to H_2_O_2_-mediated toxicity [35]. One of the deleterious effects of O_2_^**.**-^ relies on the inactivation of iron-sulfur-containing dehydratases, including the citric acid cycle enzyme—mitochondrial aconitase, through the release of the solvent-exposed iron atom from the Fe-S cluster [36]. IRP1 is a cytosolic counterpart of mitochondrial aconitase containing a fully assembled iron-sulfur [4Fe-4S] cluster, however, its biological significance as an enzyme, converting citrate into isocitrate in the cytosol is not well understood [37]. Importantly, upon iron-sulfur cluster removal, IRP1 becomes a post-transcriptional regulator of iron metabolism. By binding to IREs present in the untranslated regions (UTR) of mRNAs encoding proteins of iron metabolism, apo-IRP1 regulates their expression and thus controls iron availability in the cell. It has been reported that IRP1 is a molecular target for O_2_^**.**-^, which generates so called [3Fe-4S]-IRP1 „null” form that possesses neither aconitase nor IRE-binding activity [9]. Furthermore, our studies on mice with the genetic ablation of SOD1revealed a new,O_2_^**.**-^-dependent regulation of IRP1 leading to the strong reduction of IRP1 protein abundance [14].

Our observation of the reduction of IRP1 in SOD1 deficiency derives from the experimental model, in which the function of SOD1 is entirely abolished. In order to assess the possible influence of physiological changes of SOD1 activity on IRP1 we compared patterns of SOD1 activity and IRP1 expression levels in the liver. Although hepatic SOD1 activity varied in the range of 20 U/mg protein, IRP1 protein level changed irrespectively of these fluctuations of SOD1 activity. Similarly, significant drop of SOD1 expression/activity observed in the liver of mice heterozygous for the non-functional *Sod1* allele (*Sod1*^*+/-*^) had no impact on IRP1 level nor on IRP1 aconitase activity. These two sets of data strongly suggest that reduction in IRP1 level occurs only under conditions of severe reduction of SOD1 activity. Not surprisingly, the pattern of IRP1 protein level in the kidney of three SOD1 genotypes was the same as in the liver, except that decrease in renal IRP1 protein in *Sod1*^*-/-*^ mice is smaller compared with hepatic one. It seems that in contrast to mice with genetic IRP1 ablation [21–23], in *Sod1*^*-/-*^ mice showing only partial decline in renal IRP1, regulatory axis IRP1-HIF2α and its impact on erythropoiesis and the occurrence of polycythemia are not altered as attested by normal peripheral erythrocyte count [16].

In this study, we aimed also to verify whether the down-regulation observed in the liver of adult KO SOD1 mice occurs in the prenatal period. It is known that fetal development occurs in a state of relative hypoxia [24]. Low pO_2_ is associated with a decreased rate of cellular mitochondrial ROS production predicting lesser role of SOD1 an intracellular antioxidant. Indeed, lack of SOD1 activity has been reported not to disturb the prenatal development of mice [33,38]. In accordance, our data show also that at various stages of prenatal life, SOD1 deficiency did not affect IRP1 expression. The difference in hepatic IRP1 level between wild-type and SOD1 “null” mice appeared only postnatally as early as on day 1 *post-partum*. At birth, with the onset of breathing, arterial blood pO_2_ dramatically increases and in consequence oxygen tension shifts from relatively hypoxic *in utero* to normal in tissues [24], which requires protective activity of antioxidant enzymes including SOD1.

To obtain more insight into IRP1 regulation by reactive oxygen species, we used cellular model of mouse line RAW 264.7 cells and bone marrow-derived macrophages (BMDM) exposed to paraquat (PQ), a redox cycler stimulating production of O_2_^**.**-^ [15]. PQ is broadly used in cellular [39,40] and in *in vivo* studies [41,42] to induce O_2_^**.**-^-mediated oxidative stress. The evidence of intracellular elevation of the O_2_^**.**-^ steady-state upon the treatment with PQ is well documented by the use of spin trapping techniques [39] and biochemical methods [40]. Furthermore, PQ-induced toxicity may be prevented by SOD overexpression or administration of SOD mimetics [43,44] and PQ hypersensitivity is caused by SOD deficiency [45]. Those results emphasize the important role of O_2_^**.**-^ in PQ-mediated cellular damage.

We show here that the treatment of macrophages with PQ results in the reduction of IRP1 expression and consequently in the down-regulation of both IRP1 aconitase and IRE-binding activities. Importantly, treatment of murine B6 fibroblasts with menadione, another redox cycling drug increasing intracellular O_2_^**.**-^ level has been reported to down-regulate total IRP1 IRE binding [46], considered an indirect measure of IRP1 protein level [20]. The extent of PQ-induced regulation in macrophages was similar to that observed in the liver of KO SOD1 mice [14]. Importantly, like under SOD1 deficiency, mitochondrial aconitase expression was not affected in PQ-treated RAW 264.7 macrophages suggesting that O_2_^**.**-^-dependent regulation is only restricted to cytosolic aconitase. Likewise, sensitivity and resistance to O_2_^**.**-^ of cytosolic and mitochondrial aconitase activity, respectively has been reported in *Drosophila* displaying genetic diminution of SOD1 [42]. Although increased production of O_2_^**.**-^ is a major factor in the toxicity of PQ, it is well established that treatment with this xenobiotic gives rise to some production of hydrogen peroxide (H_2_O_2_) [15]. Extracellular H_2_O_2_was among the first factors shown to inhibit IRP1 aconitase activity and to induce IRP1 binding [10,11]. However, neither the *in vitro* treatment of cells with H_2_O_2_ [11] nor *ex vivo* experiments on the rat liver perfused with H_2_O_2_ [46] were shown to alter the expression of the *Irp1* gene. Here, we also demonstrate that exposure of RAW 264.7 cells to H_2_O_2_ has no impact on IRP1 level. Furthermore, in our recent study we showed no changes in IRP1 expression in various tissues of mice overexpressing human *SOD1* gene [47], a condition known to elevate the H_2_O_2_ steady-state level [35]. Taken together, our results allow us to conclude that O_2_^**.**-^ is a major factor responsible for the regulatory effect observed in PQ treated macrophages.

The final goal of this study was to examine the regulation of IRP1-target mRNAs (containing IRE sequences in either 5’- or 3’-UTR such as L-Ft and TfR1, respectively) in mouse BMDM treated with PQ. Importantly, we asked the question of how L-Ft is regulated by PQ in *Irp1*^*+/+*^ and *Irp1*^*-/-*^ BMDM displaying partial, PQ-induced reduction of IRP1 expression and its total, constitutive deficiency, respectively. PQ treatment was found to up-regulate L-Ft protein level in cells of both IRP1 genotypes. Keeping in mind that in cells exposed to PQ L-Ft expression was also increased at the mRNA level, we assume that the exposure of BMDM to PQ enhances the transcription of the *LFt* gene and that parallel decline in IRP1 facilitates a rise at the protein level. Our observation that L-Ft mRNA and protein levels in intact *Irp1*^*+/+*^ and *Irp1*^*-/-*^ BMDM are similar underlies the importance of transcriptional induction of *L-Ft* gene and clearly shows that IRP1 deficiency is not sufficient on its own for the elevation of L-Ft protein level.

Ferritin is an ubiquitous cytosolic protein possessing high capacity to store iron in excess of cellular needs in a soluble and non-toxic form. Ferritin protein shell is composed of 24 subunits of two types (L-Ft and H-Ft) showing different functional properties. Two mRNAs encoding ferritin subunits are uniformly regulated by IRP/IRE system [48]. It seems therefore that the H:Lratio in cells and tissues is determined by the transcriptional regulation. Of note, although the *HFt* gene is commonly considered to be transcriptionally regulated by oxidative stress [49], it is also well established that the *LFt* gene responds to oxidant agents with a mechanism that involves an upstream antioxidant responsive element (ARE) present in its promoter [49,50]. Importantly in the context of our study, transcriptional induction of the *LFt* gene has been also reported in cells treated with oltipraz, cancer chemopreventive agent [51] generating the production of O_2_^**.**-^ [52].

In conclusion, our results demonstrate that O_2_^**.**-^-dependent oxidative stress induced in PQ-treated macrophages up-regulates L-Ft transcript, reduces IRP1 protein level, and shifts the remaining pool of IRP1 to the [3Fe-4S]-IRP1 form, which is not active as a transcriptional regulator of L-Ft, nor as aconitase. As a consequence, the protein level of L-Ft, a cellular protectant against oxygen free radical-mediated damage, is up-regulated. It is plausible that in order to counterbalance the toxic effects of O_2_^**.**-^, which includes the Haber-Weiss reaction [53] and elevated free iron levels [54], cells are using O_2_^**.**-^-mediated signaling to enhance the capacity of L-Ft to sequester potentially harmful free iron. Furthermore, O_2_^**.**-^ signaling may also counteract the effect of other ROS such as H_2_O_2_, which lessen IRP1 potential to inhibit ferritin expression at the post-transcriptional level [10,11].

## Supporting information

S1 Fig Mitochondrial aconiatase (m-aco) protein level is not decreased in RAW 264.7 macrophages exposed to paraquat (PQ). RAW 264.7 cells were treated with PQ as described in the legend to Fig 3. m-aco levels were analyzed by Western blotting using mitochondrial extracts prepared from cells as described previously [55]. Samples were probed with antibody raised against purified beef heart mitochondrial aconitase kindly provided by Dr. R. B. Franklin, University of Maryland, Baltimore, MD. Results from three independent biological experiments are show.
(TIF)

S2 Fig IRP1 protein level is not decreased in RAW 264.7 macrophages exposed to hydrogen peroxide (H_2_O_2_). RAW 264.7 cells were treated with 50 μM H_2_O_2_ for 30 min, washed, resuspended in fresh medium and cultured for 6 h. IRP1 levels were analyzed by Western blotting using cytosolic extracts prepared from cells as described previously [55]. Results from three independent experiments are shown.
(TIF)
