# Supplementary figures and images for: A drastic superoxide-dependent oxidative stress is prerequisite for the down-regulation of IRP1: Insights from studies on SOD1-deficient mice and macrophages treated with paraquat
Source: PLoS One. 2017 May 19;12(5):e0176800. doi: 10.1371/journal.pone.0176800 (PMC5438123; doi:10.1371/journal.pone.0176800)

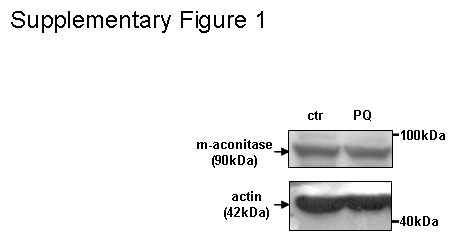

Supplement: S1 Fig — RAW 264.7 cells were treated with PQ as described in the legend to Fig 3. m-aco levels were analyzed by Western blotting using mitochondrial extracts prepared from cells as described previously [55]. Samples were probed with antibody raised against purified beef heart mitochondrial aconitase kindly provided by Dr. R. B. Franklin, University of Maryland, Baltimore, MD. Results from three independent biological experiments are show. (TIF) [file pone.0176800.s001.tif]

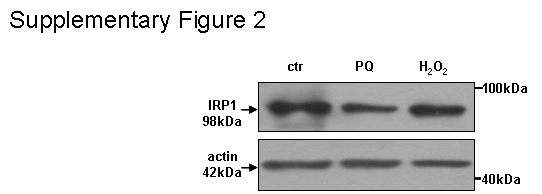

Supplement: S2 Fig — RAW 264.7 cells were treated with 50 μM H2O2 for 30 min, washed, resuspended in fresh medium and cultured for 6 h. IRP1 levels were analyzed by Western blotting using cytosolic extracts prepared from cells as described previously [55]. Results from three independent experiments are shown. (TIF) [file pone.0176800.s002.tif]
